# Signaling Regulation of FAM134‐Dependent ER‐Phagy in Cells

**DOI:** 10.1002/jcp.31492

**Published:** 2024-11-25

**Authors:** Alessandro Palma, Alessio Reggio

**Affiliations:** ^1^ Department of Biology and Biotechnologies “Charles Darwin” Sapienza University of Rome Rome Italy; ^2^ Saint Camillus International University of Health Sciences Rome Italy

**Keywords:** autophagy, endoplasmic reticulum, ER‐phagy, FAM134B, ubiquitination

## Abstract

The endoplasmic reticulum (ER) is a pivotal organelle responsible for protein and lipid synthesis, calcium homeostasis, and protein quality control within eukaryotic cells. To maintain cellular health, damaged or excess portions of the ER must be selectively degraded via a process known as selective autophagy, or ER‐phagy. This specificity is driven by a network of protein receptors and regulatory mechanisms. In this review, we explore the molecular mechanisms governing ER‐phagy, with a focus on the FAM134 family of ER‐resident ER‐phagy receptors. We discuss the molecular pathways and Posttranslational modifications that regulate receptor activation and clustering, and how these modifications fine‐tune ER‐phagy in response to stress. This review provides a concise understanding of how ER‐phagy contributes to cellular homeostasis and highlights the need for further studies in models where ER stress and autophagy are dysregulated.

## 1 Introduction

Autophagy, particularly macroautophagy, is a vital cellular process that allows cells to clear and recycle unwanted or harmful materials, such as damaged organelles, toxic protein aggregates, and invading pathogens (Reggio, Buonomo, and Grumati 2020; Klionsky 2021). This self‐sustaining mechanism relies on double‐membraned vesicles known as autophagosomes, which capture portions of the cytoplasm and deliver them to lysosomes for degradation (Abada and Elazar 2014). Within lysosomes, the breakdown of these components releases essential macromolecules that can be repurposed to fuel biosynthetic pathways, thereby maintaining cellular homeostasis (Settembre et al. 2013).

The molecular machinery driving autophagy is largely conserved across eukaryotes and involves a sophisticated network of proteins that mediate autophagosome formation, expansion, and closure (Dikic and Elazar 2018; Stolz, Ernst, and Dikic 2014). A key step in initiating autophagy is the inactivation of mTOR kinase, a major inhibitor of autophagy. When mTOR is suppressed, core autophagy proteins are recruited to specialized regions of the endoplasmic reticulum (ER) called omegasomes. At these sites, mTOR inhibition facilitates the recruitment of the Unc‐51‐like kinase 1/2 (ULK1/2) complex, which subsequently triggers phagophore nucleation by recruiting components of the class III phosphatidylinositol 3‐kinase (PI3KC3) complex I. At these nascent sites, PI3KC3 complex I catalyzes the production of phosphatidylinositol 3‐phosphate (PI3P), a lipid that attracts PI3P‐binding autophagy effectors. These effectors act as scaffolds, anchoring the ATG5–12–16 complex to the outer phagophore membrane. This complex then facilitates the conjugation of microtubule‐associated protein light chain 3 (LC3) and γ‐aminobutyric acid receptor‐associated proteins (GABARAPs) to phosphatidylethanolamine on the phagophore membranes, promoting membrane elongation and eventual closure. The final stage of autophagy, the fusion of autophagosomes with lysosomes for cargo degradation, is coordinated by a specialized protein machinery, ensuring the proper recycling of cellular materials.

## 2 Selective Autophagy and Its Significance for Cells

Autophagy was once considered a nonselective process, indiscriminately digesting various portions of intracellular material. However, over the past decade, our understanding of autophagy has evolved dramatically, revealing the existence of highly regulated, targeted forms known as selective autophagy (Gatica, Lahiri, and Klionsky 2018). This precision‐driven process is orchestrated by specialized protein effectors called autophagy receptors (Stolz, Ernst, and Dikic 2014). These receptors have the remarkable ability to recognize and bind “destruction‐tagged” cellular components, delivering them to lysosome for degradation via LC3/GABARAP protein recognition (Wilkinson 2020).

The key to this targeting lies in the receptors' LC3‐interacting region (LIR, with a consensus sequence [W/F/Y]‐X1‐X2‐[I/L/V]) or GABARAP‐interacting motif (GIM, [W/F]‐[V/I]‐X2‐V), often located within unstructured regions of the receptor proteins (Stolz, Ernst, and Dikic 2014). These motifs facilitate the direct interaction with LC3s and GABARAPs, tethering the receptors—and their bound cargo—to the autophagosomes (Stolz, Ernst, and Dikic 2014). Once recruited, both the cargo and the receptors themselves are engulfed and degraded within lysosomes (Yim and Mizushima 2020).

By employing a diverse array of autophagy receptors alongside a conserved core autophagy machinery, cells can target specific cellular or subcellular structures for removal (Wilkinson 2020). This selective process is crucial for maintaining cellular homeostasis, allowing cells to eliminate misfolded protein aggregates (aggrephagy), damaged nuclei (nucleophagy), malfunctioning mitochondria (mitophagy), excessive expanded ER domain (ER‐phagy), among others. Through selective autophagy, cells can fine‐tune their energy expenditure by focusing on one component at a time, optimizing cellular functions without the need for a wholesale breakdown of cytoplasmic material (Zaffagnini and Martens 2016).

For example, during proteotoxic stress, misfolded proteins accumulate, causing the ER to expand its membrane surface to increase its folding capacity. Once the stress subsides, cells selectively eliminate only the affected ER subdomains where these aggregates have accumulated (Cable et al. 2022; Reggio 2021; Fregno et al. 2018). This highly efficient approach allows cells to restore homeostasis while conserving energy.

However, the specificity of selective autophagy necessitates an additional layer of regulation to prevent unchecked degradation. Posttranslational modifications (PTMs), such as acetylation, phosphorylation, and ubiquitination, play critical roles in modulating the expression, localization, and activity of autophagy receptors (McEwan and Dikic 2011). These regulatory mechanisms are essential to preventing excessive and harmful autophagy, ensuring that degradation is finely controlled, even under basal conditions.

In the coming decade, a deeper understanding of these regulatory dynamics will likely pave the way for novel therapeutic breakthroughs in fields such as cancer research and genetic diseases, offering new strategies to modulate autophagy for therapeutic benefits (Klionsky 2021; Deng et al. 2017; Sharma et al. 2018).

### 2.1 ER‐Phagy: Selective Autophagy of the ER

The ER is the largest and most dynamic membranous organelle in eukaryotic cells, playing a pivotal role in lipid and protein synthesis, protein quality control, calcium and ion homeostasis, and interorganelle communication (Chen, Novick, and Ferro‐Novick 2013). Its morphology is incredibly complex, constantly reshaping in response to cellular demands (Nixon‐Abell et al. 2016). When cells fail to adapt or turn over ER membranes efficiently, they struggle to meet these demands, potentially leading to the exhaustion of essential functions (Ferro‐Novick, Reggiori, and Brodsky 2021).

To regulate ER membranes and subdomains, cells rely on a highly selective form of autophagy known as ER‐phagy (Chino and Mizushima 2020; Grumati, Dikic, and Stolz 2018; Gubas and Dikic 2022). This process ensures the degradation of specific portions of the ER, including lipid membranes and protein aggregates that accumulate in the ER. Like other forms of selective autophagy, ER‐phagy is driven by autophagy receptors harboring LC3‐interacting regions (LIR domains), which serve as bridges between cargo and autophagosomal membranes (Stolz, Ernst, and Dikic 2014).

Currently, 11 distinct ER‐phagy receptors have been identified, each contributing to different aspects of ER maintenance (Mochida and Nakatogawa 2022). These receptors are essential for basal ER turnover, membrane reshaping in response to stress, and the lysosomal degradation of protein aggregates within the ER. The diversity of ER‐phagy receptors highlights a complex network of adaptors, each specialized to target specific subdomains of the ER or to manage stress responses in distinct cell types (Wilkinson 2019). Moreover, ER‐phagy largely participate to the direct removal of ER subdomains where misfolded proteins accumulate when fail to properly engage the ER‐associated degradation (ERAD) machinery. Contextually, ER‐phagy receptors may cooperate with adaptor proteins (e.g., CALNEXIN and PGRMC1) to isolate while favoring vesiculation of such domains for their incorporation of LC3/GABARAP‐decorated autophagosomes (Rudinskiy and Molinari 2023).

Among these receptors, FAM134B stands out as the most well‐characterized (Reggio 2021; Khaminets et al. 2015). It is part of a larger family of ER‐resident proteins that share similar domain composition and function, marking the first identified family of ER‐phagy receptors (Reggio 2021). Recent research has uncovered the upstream signaling pathways that regulate FAM134B and revealed how PTMs play a critical role in modulating FAM134B‐dependent ER turnover (Reggio 2021; Foronda et al. 2023; González et al. 2023; Di Lorenzo et al. 2022; Berkane et al. 2023; De Leonibus et al. 2024; Wang et al. 2023).

This review synthesizes these recent discoveries, providing a clear and comprehensive understanding of the molecular mechanisms underlying ER‐phagy and the regulation of its key players. By unraveling these complexities, we can better appreciate how cells maintain ER homeostasis and respond to stress through selective autophagy.

## 3 The FAM134 Family Members

FAM134A, FAM134B, and FAM134C are intra‐membrane ER‐resident proteins that play a prominent role in the basal turnover of ER sheets (Reggio 2021; Khaminets et al. 2015). These proteins harbor specific domains that enable them to mediate this process (Reggio 2021):

- LIR domain: located at the c‐terminus of these proteins. It is responsible for their interaction with LC3 on newly formed phagosome membranes.
- Reticulon homology domain (RHD): the domain anchors these proteins to the ER while giving them the ability to curve the ER membranes.

**Figure 1:**
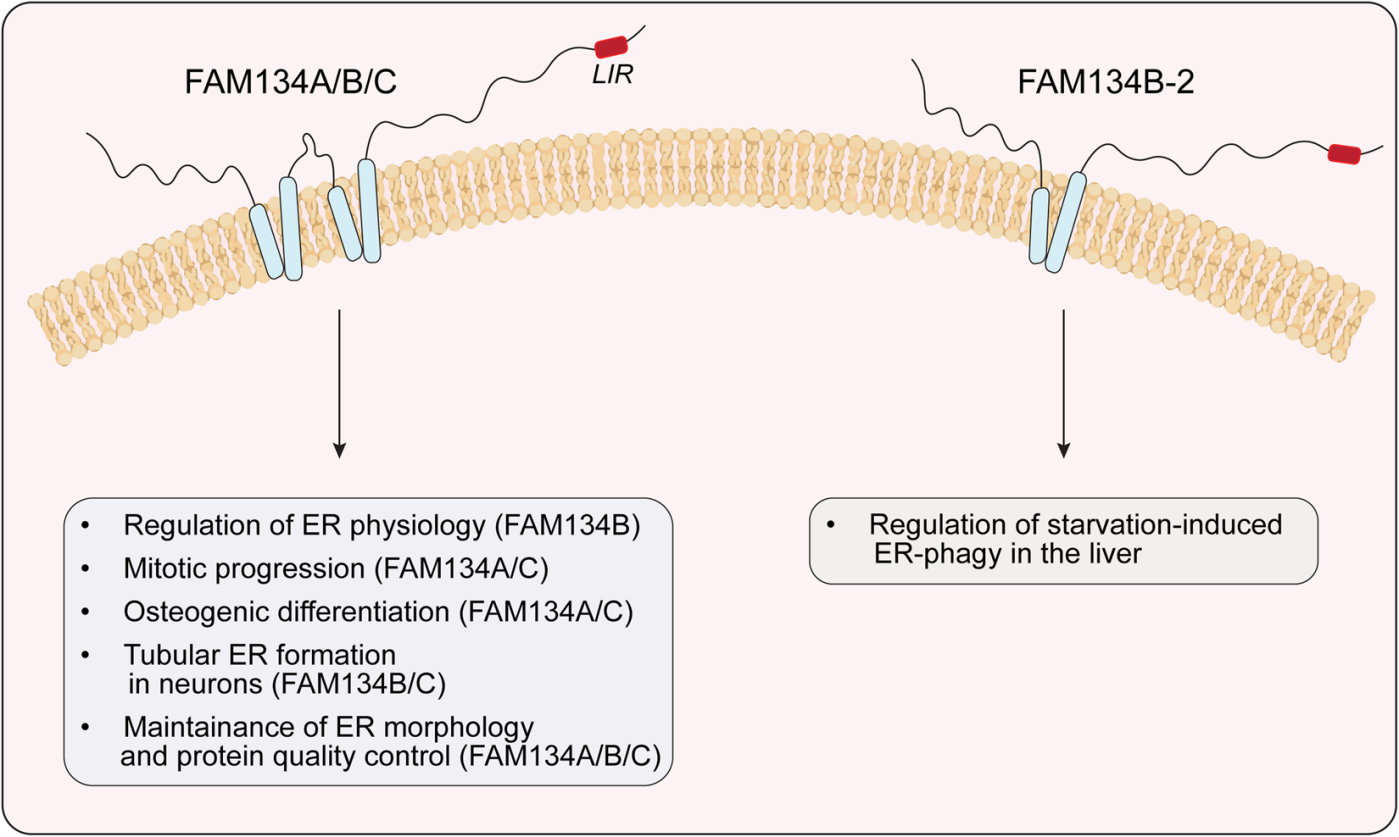
Roles of FAM134 protein family. The figure shows the most important roles of FAM134A, B and C (left), as well as the recently identified truncated form of FAM134B (FAM134B‐2, right). The LC3‐interacting region (LIR) domain is highlighted in red.

Among the three members, FAM134B is the most extensively studied receptor, and a substantial body of evidence supports its key role in maintaining cell homeostasis through the regulation of proper ER physiology (Figure 1). Alterations or mutations that affect FAM134B expression have been implicated in several human diseases, including esophageal and colorectal cancers (Haque et al. 2016; Kasem et al. 2014), as well as in the degeneration of primary dorsal root ganglion neurons, leading to sensory and autonomic neuropathy (HSANII) (Foronda et al. 2023; Kurth et al. 2009; Hübner and Dikic 2020). Recently, a shorter isoform (*N*‐terminally truncated version) of FAM134B, has been characterized. This FAM134B‐2 is highly expressed in liver and largely contribute to mediate ER‐phagy in hepatocytes upon nutrient stress (Kohno et al. 2019).

Conversely, FAM134A and FAM134C are less well understood. FAM134A has been associated with mitotic progression (Toyoda et al. 2017) and is targeted by hsa‐miRNA940 in the regulation of osteogenic differentiation (Hashimoto et al. 2018). FAM134C has been reported to regulate neurite outgrowth in human neuroblastoma cells and primary rat hippocampal neurons (Wang et al. 2013). Moreover, FAM134C intimately collaborates with FAM134B to properly form tubular ER in the axon of motor and sensory neurons (Iavarone et al. 2024).

Recent research efforts have clarified the functions and roles of these proteins, as well as systems to manipulate FAM134‐dependent ER‐phagy, significantly enhancing the clinical value of a deeper understanding of ER biology (Foronda et al. 2023; González et al. 2023; Berkane et al. 2023; De Leonibus et al. 2024; Jiang et al. 2020).

All three FAM134 proteins are essential for maintaining ER morphology and protein quality control (Reggio 2021). The absence of any FAM134 family member leads to abnormal ER expansion, misfolded protein accumulation, and heightened ER stress (Reggio 2021; Fregno et al. 2018; Khaminets et al. 2015; Forrester et al. 2019). Restoration of these proteins reverses this process by promoting ER fragmentation and lysosomal degradation (Reggio 2021). In contrast, mutations in the LIR domain that disrupt its autophagic function negatively limit ER‐phagy, underscoring the importance of this interaction in the degradation pathway (Reggio 2021). Interestingly, while all three proteins are crucial for ER homeostasis, their expression levels vary across tissues and cell types, suggesting they may have distinct, context‐dependent roles (Reggio 2021). For example, FAM134C works synergistically with FAM134B to facilitate the degradation of misfolded Collagen I, while FAM134A appears to mediate a FAM134B‐ and LIR‐independent degradation pathway (Reggio 2021; Berkane et al. 2023).

### 3.1 Ubiquitination

**Figure 2:**
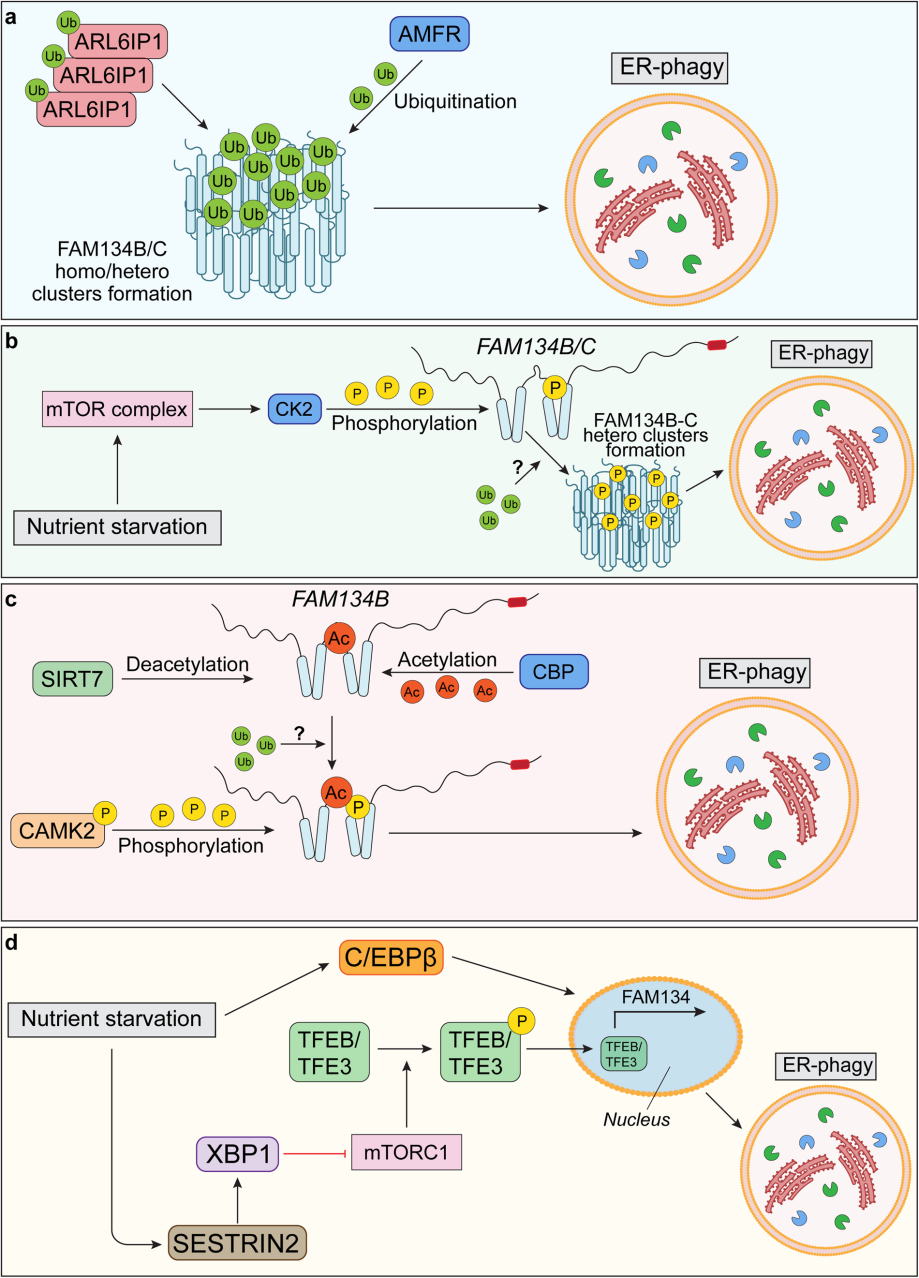
Signaling pathways converging on FAM134B promote its clustering into homomeric and heteromeric protein complexes. (a) The ubiquitination mechanism of FAM134B/C leads to homo/hetero cluster formation that drives ER‐phagy. (b) Imbalances in nutrient availability serve as critical stimuli that trigger the massive expression of FAM134B. In this context, CK2 phosphorylates the ER‐resident FAM134B at specific residues within the RHD domain, marking the protein for subsequent ubiquitination by AMFR. In its activated form, FAM134B nucleates to form heteromeric protein clusters. (c) Acetylation serves to prime FAM134B for phosphorylation and subsequent modulation of ER‐phagy. (d) Transcriptional mechanism of ER‐phagy regulation induced by the modulation of SESTRIN2 and TFEB/TFE3 nuclear translocation. ER, endoplasmic reticulum; RHD, reticulon homology domain.

A variety of proteins have been identified as crucial players in ER fragmentation and ER‐phagy under specific conditions (Jiang et al. 2020; Bhaskara et al. 2019; Siggel et al. 2021). It is increasingly evident that multiple pathways, along with PTMs, interact dynamically and sometimes complementarily to modulate ER‐phagy (Figure 2a) (Foronda et al. 2023; González et al. 2023; Di Lorenzo et al. 2022; Berkane et al. 2023; Forrester et al. 2019). While certain relationships between these pathways and receptors remain to be fully uncovered, our understanding of the molecular signals and PTMs that activate FAM134B, FAM134A, and FAM134C has grown significantly.

One of the most important PTMs in this context is ubiquitination, a reversible modification that regulates the activity and turnover of cellular proteins. Recent studies have revealed that the ubiquitination of FAM134 paralogues plays a critical role in spatially controlling their membrane‐bending capabilities and overall activity (Foronda et al. 2023; González et al. 2023). High‐resolution proteomics has identified key ubiquitination sites—K90, K160, K264, and K247—within the RHD of FAM134B. When ER‐phagy is induced, ubiquitination levels at these sites increase, and pharmacological inhibition of the ubiquitin‐activating enzyme blocks both FAM134B ubiquitination and ER‐phagy (González et al. 2023).

Ubiquitination prompts structural rearrangements in the RHD domains, enabling interactions between neighboring RHD‐containing proteins—a prerequisite for inducing membrane curvature and fission (González et al. 2023; Bhaskara et al. 2019; Siggel et al. 2021). Within these high‐density protein clusters, reticulons (RTNs), mammalian ATG8 proteins, and components of the ubiquitin machinery are present (Foronda et al. 2023; González et al. 2023). Their presence suggests active involvement in two critical processes: (i) the structural remodeling of ER regions and (ii) the pinching off of ER portions during ER‐phagy (Bhaskara et al. 2019; Siggel et al. 2021). Notably, the E3 ligase AMFR has been found in FAM134B‐rich clusters and has been confirmed as the enzyme responsible for catalyzing FAM134B ubiquitination (González et al. 2023).

Another RHD‐containing protein involved in forming these FAM134B clusters is ARL6IP1. Like FAM134B, ARL6IP1 undergoes ubiquitination and forms both homomeric and heteromeric protein clusters, interacting with FAM134B, FAM134A, and FAM134C. ARL6IP1 plays a significant role in ER‐phagy and maintaining ER homeostasis (Foronda et al. 2023). In Arl6ip1‐deficient mice, primary cells show incomplete ER membrane budding and impaired ER‐phagy flux (Foronda et al. 2023). These mice exhibit expanded ER sheets in sensory neurons, which degenerate over time (Foronda et al. 2023). In humans, mutations in ARL6IP1 are linked to SPG61, a neurodegenerative disorder characterized by progressive leg spasticity and increased sensitivity to pain—symptoms that overlap with those of hereditary sensory and autonomic neuropathy (HSAN), a condition caused by FAM134B loss‐of‐function mutations (Foronda et al. 2023; Kurth et al. 2009).

### 3.2 Phosphorylation

While ubiquitination promotes the clustering of FAM134 proteins, phosphorylation serves as the crucial signal that triggers this process (Figure 2b). Specifically, phosphorylation within the RHD of these proteins signals the ubiquitin machinery to target them for modification, facilitating their role in ER‐phagy (Reggio 2021; Di Lorenzo et al. 2022; Jiang et al. 2020). This posttranslational modification is regulated by the mTOR pathway, involving downstream kinases such as ATR, ATM, Chk1, Akt, and CK2 (Berkane et al. 2023). Among these, CK2 has been experimentally shown to phosphorylate both FAM134B and FAM134C at key serine residues within their RHD domains (Di Lorenzo et al. 2022; Berkane et al. 2023). For example, mutation of FAM134C at S258A significantly impairs ER‐phagy, while mutations at S149A, S151A, and S153A similarly hinder mTOR‐dependent ER‐phagy (Berkane et al. 2023). Consistently, loss of CK2 activity—either through genetic knockout or pharmacological inhibition—blocks ER‐phagy induction following Torin1 treatment in cells overexpressing FAM134B and FAM134C (Berkane et al. 2023).

Phosphorylation not only marks these receptors for ubiquitination but also facilitates their organization into high‐density heteromeric FAM134B‐FAM134C clusters (Berkane et al. 2023). These clusters are essential for driving membrane curvature and subsequent ER fragmentation (Jiang et al. 2020; Bhaskara et al. 2019; Siggel et al. 2021). Inhibiting E1 (ubiquitin‐activating enzyme) or CK2 activity, or genetically altering the target serine residues, prevents ubiquitination and the formation of these clusters, effectively halting ER‐phagy (Berkane et al. 2023).

Interestingly, FAM134C can be phosphorylated by CK2 even under basal conditions, in the absence of ER‐phagy stimuli (Berkane et al. 2023). This “primed” state suggests that FAM134C may act as a nucleation point for the formation of FAM134B‐FAM134C clusters (Berkane et al. 2023), aligning with previous observations that FAM134C resides in a relatively inactive state under normal conditions (Reggio 2021). However, upon proteotoxic stress, it enhances FAM134B‐dependent ER‐phagy, amplifying the degradation process (Reggio 2021).

Phosphorylation also appears to influence FAM134C's interaction with LC3. CK2 has been shown to phosphorylate residues near the LIR domain of FAM134C (S435, S436, and T440), which may either inhibit or enhance the binding of FAM134C to LC3, depending on the experimental context (Di Lorenzo et al. 2022). This conflicting evidence suggests a nuanced role for phosphorylation in regulating ER‐phagy, possibly influenced by additional, yet unknown factors interacting with the LIR domain.

Phosphorylation also plays a pivotal role in FAM134B oligomerization (Jiang et al. 2020), a key event required for FAM134B‐mediated ER‐phagy. In addition to CK2, CAMK2B phosphorylates FAM134B at S151 in the RHD domain, further promoting the formation of FAM134B‐rich clusters, which drive ER membrane fragmentation and ER‐phagy (Jiang et al. 2020).

### 3.3 Acetylation

Recent discoveries have shown that FAM134B undergoes acetylation by CBP acetyltransferase, a modification that significantly enhances ER‐phagy (Wang et al. 2023) (Figure 2c). Acetylation of FAM134B increases CAMK2‐mediated phosphorylation, amplifying its role in promoting ER degradation via the lysosomal pathway (Wang et al. 2023). In contrast, SIRT7 counteracts this process by deacetylating FAM134B, tempering its activity to prevent excessive ER breakdown and maintain cellular balance (Wang et al. 2023).

However, it remains unclear whether additional PTMs—such as ubiquitination—might follow CBP‐dependent acetylation or CAMK2B‐dependent phosphorylation to further regulate FAM134B's ability to form clusters and modulate its activity. The interplay between these PTMs could provide a multi‐layered control mechanism, refining how FAM134B orchestrates ER‐phagy.

Although more research is needed to uncover the full range of functions behind these supramolecular clusters, it is evident that the high‐density homomeric and heteromeric clusters formed by FAM134B are critical sites of action. These clusters act as hubs where ER membrane fission and the incorporation of ER‐derived vesicles into nascent autophagosomes occur (Khaminets et al. 2015; Bhaskara et al. 2019), ensuring efficient and selective degradation of ER components.

### 3.4 Transcriptional Circuits

ER‐phagy is not solely governed by PTMs; it is also tightly regulated at the transcriptional level (Figure 2d). A key regulatory pathway involves the MiT/TFE transcription factors (Cinque et al. 2020), which modulate ER‐phagy through the SESTRIN2‐XBP1 axis (De Leonibus et al. 2024). In response to nutrient stress, SESTRIN2 activates XBP1, which in turn inhibits mTORC1‐dependent phosphorylation of TFEB and TFE3. This inhibition allows TFEB and TFE3 to translocate into the nucleus, where they activate the expression of FAM134B, a critical receptor in ER‐phagy (De Leonibus et al. 2024; Cinque et al. 2020). This nutrient‐sensitive signaling network plays an essential role in tissue development and organismal growth by fine‐tuning ER‐phagy in response to environmental changes (Forrester et al. 2019; Cinque et al. 2020).

In parallel, the transcription factor C/EBPβ contributes to ER‐phagy regulation, particularly during starvation. C/EBPβ promotes the expression of a short isoform of FAM134B, FAM134B‐2 (Kohno et al. 2019), which specifically drives starvation‐induced ER‐phagy in the liver (Kohno et al. 2019). Similarly, the transcriptional activation operated by MEF2D‐NR4A1 also drives FAM134B‐2 expression, indicating the existence of converging systems that may control the expression to respond to different energy demands in cells (Shiozaki et al. 2022).

These transcriptional mechanisms work in concert, increasing the abundance of FAM134B and other ER‐phagy receptors, priming them for downstream modifications—such as phosphorylation and ubiquitination—required for full activation and function in ER‐phagy.

Together, these regulatory circuits ensure that ER‐phagy is dynamically adjusted to the cell's needs, providing a balance between ER turnover and maintaining cellular homeostasis during nutrient fluctuations and stress.

## 4 Conclusion

The intricate regulation of ER‐phagy highlights the remarkable adaptability of cells in maintaining homeostasis under various stress conditions. PTMs, such as ubiquitination, phosphorylation, and acetylation, converge to fine‐tune the activity of key ER‐phagy receptors, particularly members of the FAM134 family. These modifications regulate not only receptor activation but also their assembly into functional clusters, orchestrating the selective degradation of ER components. Simultaneously, transcriptional circuits ensure a timely response to environmental cues, linking nutrient sensing and cellular stress to the upregulation of the ER‐phagy machinery.

This multilayered regulation safeguards ER integrity and demonstrates how cellular recycling systems can be tightly controlled to balance survival and growth. Understanding these regulatory dynamics opens new avenues for therapeutic intervention, particularly in diseases where ER stress and autophagy play critical roles. In the coming decade, groundbreaking therapeutics will likely leverage perturbagens capable of modulating one or more of these regulatory steps to restore ER function and proper cell physiology.

## Conflicts of Interest

The authors declare no conflicts of interest.

## Data Availability

The authors have nothing to report.
